# Using the Burning of Polymer Compounds to Determine the Applicability of the Acoustic Method in Fire Extinguishing

**DOI:** 10.3390/polym16233413

**Published:** 2024-12-04

**Authors:** Valentyna Loboichenko, Jacek Lukasz Wilk-Jakubowski, Alexander Levterov, Grzegorz Wilk-Jakubowski, Yevhenii Statyvka, Olga Shevchenko

**Affiliations:** 1 Department of Civil Security, Lutsk National Technical University, Lvivska St., 75, 43000 Lutsk, Ukraine; vloboichm@gmail.com; 2 Dpto. de Ingeniería Energética, Escuela Técnica Superior de Ingeniería, Universidad de Sevilla, Camino de los Descubrimientos s/n., 41092 Sevilla, Spain; 3 Department of Information Systems, Kielce University of Technology, 7 Tysiąclecia Państwa Polskiego Ave., 25-314 Kielce, Poland; 4 Department of Management and Organization of Activities in the Field of Civil Defense, National University of Civil Defence of Ukraine, Chernyshevska Str., 94, 61023 Kharkiv, Ukraine; alionterra@gmail.com (A.L.); jekas657@gmail.com (Y.S.); shevchenkoolga2008@gmail.com (O.S.); 5 Institute of Internal Security, Old Polish University of Applied Sciences, 49 Ponurego Piwnika Str., 25-666 Kielce, Poland; grzegorzwilkjakubowski@wp.pl; 6 Institute of Crisis Management and Computer Modelling, 28-100 Busko-Zdrój, Poland

**Keywords:** polymer, organic compound, combustion, applied acoustics, electrical engineering, acoustic method, smoke-filled area, environmentally friendly method, monitoring, fire safety, security

## Abstract

In order to achieve the objective of the work—an experimental study of the capabilities of the acoustic method for extinguishing organic compounds and for ensuring environmental monitoring—the effect of the combustion of various polymers on the acoustic parameters of the medium is considered. The negative effect of the combustion of organic substances on the medium is noted. The features of the use of fire extinguishing agents are analyzed, and it is noted that the acoustic method is a promising, inexpensive, and environmentally friendly approach for use in extinguishing fires. The ideas about the capabilities of this method using the combustion of various polymers were further developed, which is the novelty of the work. As the main results, it is proposed to use the angle of incidence, concentration of suspended particles, temperature, and wave resistance of the environment as special correction factors for acoustic sensors when monitoring in a smoky space. The possibility of using the combustion parameters of organic compounds to determine the properties of acoustic waves in a smoke-filled area is shown. The perspective of implementing the results obtained in the practice of fire prevention and liquidation was observed to increase the efficiency of fire extinguishing and increase the safety of the population and personnel of special services. The proposed approach can become part of the ecological and economic innovations of municipal communities and national strategies to achieve the goals of sustainable development.

## 1. Introduction

Significant changes in the natural environment that have occurred in recent decades have contributed to the emergence of a new paradigm of human interaction with the environment, which includes the achievement by 2030 of the 17 ambitious goals of sustainable development proposed by member countries of the UN (United Nations) [1]. Among others, they include the fight against climate change, the use of renewable energy, the preservation of marine and terrestrial ecosystems, including the transition to carbon-free energy carriers [2] and renewable energy sources [3], and the reduction of negative human impacts on natural ecosystems [4]. The use of a significant amount of organic polymer substances used in industry, agriculture, and the daily life of the population is the result of the scientific and technical development of the modern world [5,6,7,8,9,10,11,12]. The generation of waste from their use is one of the main problems facing humanity today [13]. The consequence of the generation of this waste is a modern global environmental problem: the microplastic pollution of the environment [14,15]. It is expected that by 2050, more than 590 million tons of plastic will be produced on our planet [16]. The amount of microplastics formed during this time will be 120 million tons [14]. Even now, the concentration of microplastics in some soils can exceed the concentration in the ocean by 23 times [14]. Microplastic particles easily penetrate cellular barriers into living organisms and cause cytotoxicity, metabolic disorders, and DNA damage. They can accumulate in the stomach, lungs and placenta of humans, negatively affecting female fertility [15]. At the same time, the negative impact of organic polymer compounds is not limited to physical effects. There are both the chemical pollution of the environment [16] and negative toxicological effects [17,18] on living organisms. More than 9 million tons of plastic waste that enters the ocean contributes to the distribution of microplastics in all layers of water. Due to its high bioavailability, it enters marine organisms, causes neurotoxic effects, disrupts metabolism, and affects their behavioral functions and development [17,18]. The thermal decomposition of organic polymer compounds due to fires can also be the cause of environmental pollution due to the additional formation of pollutants—combustion products. Consequently, an urgent issue is the study of the peculiarities of the liquidation of such fires, both to minimize the consequences for the environment and to reduce the risks to the life and health of special services specialists and the population.

### 1.1. Features of Fires of Organic Compounds

The most well-known and widespread organic compounds include plastic, wood, textiles, paper, and oils; they can also be part of various materials and complex mixtures. For example, wood is a mixture of cellulose, hemicellulose, and lignin, all of which are polymers [19]. The composition of the material plays a key role. Paper mainly contains an organic polymer, such as cellulose, but may contain additional substances used in its production (pigments, plasticizers, softeners, dyes, etc.) [20,21]. Plastic can be a mixture of polymeric organic compounds with different chemical formulas and structures, which influences the combustion process [22]. Styrofoam is expanded polystyrene [23], where textiles are a mixture of natural and synthetic fibers (cellulose, polypropylene, polyamide, etc.) [24]. 

The combustion of organic polymer materials, such as plastic, polystyrene, textiles, wood, and paper, which mainly represent a fire load in closed environments, is accompanied by the decomposition of substances and the formation of a wide range of chemical products. They change the physical properties of the environment and represent a significant danger [25]. In particular, there is an increase in temperature and a change in the chemical composition and density of the environment, which are caused by the products of the thermal destruction of the polymer compounds.

#### 1.1.1. Features of Combustion of Organic Substances

In the process of the combustion of organic polymer compounds, the formation of various final products is possible [26,27]. So, for example, in the general case, the burning of plastic (polyethylene and polypropylene) is accompanied by the formation of such substances as carbon dioxide, water, carbon monoxide, methane, and soot. The chemical equation will have the form (1):

(1) $$(C_{2}H_{4}{)}_{n}+O_{2}\to CO_{2}+H_{2}O+CO+CH_{4}+C$$

During combustion, polyurethane emits carbon dioxide, water, carbon monoxide, and ammonia, according to (2):

(2) $$(C_{3}H_{7}NO_{2}{)}_{n}+O_{2}\to CO_{2}+H_{2}O+CO+NH_{3}$$

For natural textiles, for example, cotton (90% consists of cellulose), the combustion products include carbon dioxide, water, carbon monoxide, methane, and formaldehyde according to (3):

(3) $$(C_{6}H_{10}O_{5}{)}_{n}+O_{2}\to CO_{2}+H_{2}O+CO+CH_{4}+HCCO$$

During the combustion of mixtures of organic and inorganic compounds, processes of the simultaneous thermal decomposition of these compounds occur. For example, for plasterboard, the main process is the dehydration of gypsum CaSO_4_⋅2H_2_O. Gypsum contains two molecules of crystallization water that are released during heating or burning. During this process, gypsum is transformed into anhydride, which is accompanied by the release of water vapor, according to (4):

(4) $$CaSO_{4}\cdot 2H_{2}O\to CaSO_{4}+2H_{2}O$$

The combustion of paper (cardboard, wood, and cellulose) leads to the formation of carbon dioxide, carbon monoxide, methane, soot, formaldehyde, and acetaldehyde according to (5):

(5) $$(C_{6}H_{10}O_{5}{)}_{n}+O_{2}\to CO_{2}+H_{2}O+CO+CH_{4}+HCCO+CH_{3}CHO+C$$

For engine (motor) oils consisting of a mixture of hydrocarbons, during combustion, carbon dioxide, water, carbon monoxide, and soot (6) are formed, as well as the formation of sulfur gas and unburned hydrocarbons:

(6) $$C_{x}H_{y}+O_{2}\to CO_{2}+H_{2}O+CO+C+\mathrm{unburned}\;\mathrm{hydrocarbons}$$

At the same time, it should be noted that depending on the type of combustion (complete or incomplete) and temperature, with or without sufficient ventilation, etc., the chemical composition and output of one or another combustion product will vary significantly [28,29]. Under the conditions of the presence of impurities in the polymer, the products of incomplete combustion may contain various additional halogens, phosphorus, nitrogen derivatives, etc. In addition, incomplete combustion products such as xylenes, naphthalene, styrene, chloromethane, etc., may occur [30]. At the same time, it is noted that natural organic products release fewer polycyclic aromatic hydrocarbons than synthetic plastics.

The peculiarities of the course of fires of organic polymer compounds in closed rooms lead to the formation of various dangerous combustion products at different stages of the fire (smoldering combustion, early flame that is well ventilated, and fully developed flame that is not sufficiently ventilated). Due to the temperature regimes, the high speed of the reactions, and the scale, the last stage is characterized by the highest yield of toxic compounds [31].

Since organic compounds, including polymers, are known to have low fire resistance [32,33,34,35], various compounds are added or various coatings are used to increase their resistance to heat. Thus, various additives to improve the properties of polymer compounds also affect their combustion processes. The use of admixtures of flame retardants and clay nanoparticles to improve the fire resistance of plastics, using the example of polypropylene and polyamide, showed the effect of these admixtures on the growth of CO emissions and the size of soot particles when burning these materials [36]. When studying the combustion of polymer compounds used in shipbuilding, in the stage of primary pyrolysis, the matrix–polymer resin (polyvinyl ether composite, polyethylene, or aramid) is burned, while the reinforcement fibers (E-glass) remain stable [37]. The addition of polyhedral oligomeric silsesquioxanes to the matrix of silicone rubber (the combustion products of which are toxic) does not significantly affect the overall increase in the mass of gaseous products, although it increases the concentration of cyclic siloxanes in the reaction products [38].

#### 1.1.2. Consequences of the Combustion of Organic Substances on the Environment

Undoubtedly, uncontrolled spontaneous phenomena, such as fires of organic substances, lead, in consequence, to significant material and immaterial losses, threatening the health and lives of humans and animals, including the entire ecosystem. As an exemplification of the negative impact on the environment, the fact that about 20% of total CO_2_ emissions into the Earth’s atmosphere are a consequence of fires can be used. Nowadays, fire protection uses, depending on the class of fire, various chemicals or liquids to interrupt the combustion reaction [39,40].

At the same time, the burning of various polymer compounds contributes to the appearance of additional dangerous compounds in the environment. The presence in their structure of a large number of repeating units (several tens–several thousands, etc.) of a monomer product, which can also have a complex structure (ether, acid, alkyl, etc.), the presence of aromatic, double, and triple bonds, and atoms of inorganic components (nitrogen, sulfur, halogens, phosphorus, etc.), depending on the combustion conditions, will contribute to the rupture of the polymer chain in different places and the products of their incomplete oxidation can vary significantly. Accordingly, it is possible to form a larger number of pollutants, as well as products of their further interaction with each other. Another risk factor is the presence in polymers of auxiliary substances, such as fire retardants, plasticizers, dyes, etc.; substances that determine the characteristics of this type of polymer plastic. Their combustion will also contribute to the formation of additional pollutants. The prevention of combustion and the rapid extinguishing of fires of these substances is one of the ways to prevent the release of their decomposition products into the environment, and thus help to minimize its pollution. 

The main toxic compounds are carbon oxides, hydrogen cyanide, hydrogen chloride, nitrogen oxides, sulfur dioxide, ammonia, phenol, some aldehydes, and polycyclic aromatic compounds [41]. In this context, research on the influence of various components on the process of extinguishing flames plays an important role [37,38,39,40,41]. Table 1 presents the impact of some combustion products of organic matter on humans and the environment. 

Different polymer compounds are characterized by the release of individual toxic substances. In particular, the burning of polymers such as low-density polyethylene, polymethyl methacrylate, and polystyrene is characterized by the toxic effect of various amounts of CO, depending on the temperature and burning conditions. While for polyamide-6,6, the main contribution to toxicity is made by hydrogen cyanide, and additionally, due to carbon and nitrogen oxides, when burning polyvinyl chloride, hydrogen chloride makes the biggest contribution to the toxic effect. The combustion of tire rubber, which is a complex mixture of natural (isoprene) rubber, synthetic (styrene–butadiene rubber, butyl rubber) rubber, and vulcanizing agents, contributes to the appearance in the environment of dangerous compounds, such as solid particles, carbon oxide, sulfur oxides, nitrogen oxides, polycyclic aromatic hydrocarbons, dioxins, furans, hydrogen chloride, benzene, polychlorinated biphenyls, and heavy metals (cadmium, nickel, zinc, mercury, chromium, and vanadium), and volatile organic compounds [49].

Dangerous compounds entering the environment during fires of organic materials, such as furniture, transformers, and electronics (for example, chlorinated and brominated dibenzo-p-dioxins and dibenzofurans, hexachlorobenzene, perchlorinated chemicals, etc.), can also accumulate in the bodies of firefighters, causing the appearance of cancer [50]. Some organic compounds released during fires can also accumulate in the personal protective equipment of firefighters [51].

As already mentioned above, one of the ways to reduce the negative impact on the environment and people during the burning of organic synthetic compounds, including polymeric compounds, is the addition of flame retardants—substances that increase their resistance to fire [52,53,54]. It should be taken into account that the composition of flame retardants (for example, the presence of nitrogen-, phosphorus-, and halogen-containing compounds [55]) is an additional factor in the formation of dangerous toxic compounds. For example, bicyclophosphate ethers, carbonyl fluoride, hydrogen fluoride, perfluoroisobutylene, polychlorinated biphenyls, halogenated dioxins, and furans that enter the environment can accumulate and cause additional negative effects on living beings [56,57]. Another way is to use effective methods and methods of fire extinguishing with the use of means that allow you to quickly identify the danger, eliminate the source of ignition, ensure the safety of specialists, etc.

### 1.2. Features of Extinguishing Fires

For the fire extinguishing of organic compounds in a solid or liquid state, a set of organizational, methodological, and technical approaches is used, with the use of various means of fire extinguishing.

Therefore, aerial firefighting equipment (helicopters or airplanes) [58], cars [59], stationary systems [60], and robotic systems [61] can be used to extinguish forest fires. To prevent the occurrence of fires, calculated forecasting methods and models are used [62,63], as is an intelligent fire detection and forecasting approach [64,65].

One of the common means of extinguishing such fires is water, which can be supplied in a finely atomized state or under lower pressure (larger drops) [66,67]. To improve the fire extinguishing characteristics of water, special additives of organic or inorganic substances can be added to it [68,69]. Various fluorine-containing or fluorine-free foams are also used [70,71,72], additional compounds that improve the characteristics of these foams [73,74]. Alternative compounds are also used, such as gel systems [75,76], siloxanes [77], nanoscale aerogel fire-extinguishing agents [78], and others.

At the same time, these compounds, together with combustion products, can also make a negative contribution to the pollution of water bodies and soils and cause ecotoxicological effects on the biota.

It is noted that even when burning the same group of substances (a model living room), depending on the method of fire extinguishing, the formation of different substances or variation in their concentration depending on the chosen method of fire extinguishing is likely. In particular, finely atomized water (under high pressure) contributed to the reduction in contaminants, while the use of foams and water under low pressure increased the appearance of contaminants [79].

The inconveniences of using conventional firefighting methods include the cost of purchasing them, the short duration of their operation, and the adverse impact on the surrounding environment (as a result, halon extinguishers are phased out of production, and their use is being prohibited on the basis of the provisions of the Geneva Convention on the prohibition of gases that affect the formation of the ozone hole). In the case of conventional firefighting techniques, the rapid replenishment of the extinguishing agent is, in practice, impossible, as it would involve the need to transport it quickly to the site of the fire and fill the tank by authorized qualified services. This is of considerable importance in the event of a fire, when any delay in time affects the level of the material and immaterial losses incurred. For this reason, in recent years, there has been a growing need to explore new, more sophisticated, and environmentally friendly firefighting techniques.

### 1.3. Features of the Application of the Acoustic Method for Detecting and Extinguishing Fires of Organic Substances

A separate issue in the case of the use of classical fire protection solutions is the necessity of subjecting tanks containing the extinguishing agent to temporary pressure tests, which is not required in the case of the use of the acoustic technique (due to its use of the natural mechanisms of the propagation of acoustic waves for extinguishing flames). In this context, many features of the application of the acoustic method to detect and extinguish fires of organic substances acquire special significance. 

The use of acoustic waves appears not only as an effective method for extinguishing flames [80,81,82], but also as a method that is safer, cheaper to operate, and non-invasive to the environment, unlike classical firefighting agents. This arises from the absence of the chemical nature of acoustic waves, the lack of emission of harmful pollutants into the environment, and the absence of the formation of difficult-to-remove stains and deposits on the extinguished objects, which are indispensable for classical firefighting agents. Since water or chemicals are not used when extinguishing flames, the undoubted advantage of acoustic technology is its non-invasive nature in areas that are intolerant of water or where there is a high risk of damaging room components when using conventional fire extinguishing agents. As acoustic energy is clean (zero-waste technology) and leaves no environmentally harmful by-products, this is a major advantage. Furthermore, once the flames have been extinguished, one can quickly return to normal operations, which distinguishes this method from the techniques currently applied [83].

It is worth noting that acoustic fire extinguishers can use machine learning algorithms to detect flames (especially deep learning as a subdiscipline of machine learning). This integration of acoustic technology with modern vision techniques using, for example, deep neural networks (DNNs) allows one to increase the effectiveness of fire ignition extinguishing, not allowing it to spread, and to reduce the response time to a minimum (early response systems) [84]. Furthermore, information on flame detection can be immediately forwarded to relevant services. In practice, the effectiveness and speed of flame and smoke detection depend on the algorithms applied. In the case of embedded systems, based on the analysis performed, the modifications of the neural network and transfer learning, the MASK R-CNN (Region-based Convolutional Neural Network) and SSD (Single Shot Detector) MobileNet models have been shown to be highly effective [85]. Among other things, Mask R-CNN can be used to solve data segmentation problems by separating objects in videos or images. For SSD MobileNet, a high detection speed was achieved at the cost of recognition accuracy to find small objects, while Mask R-CNN recorded an approximately three times longer object detection time (this network can be successfully applied to detect multiple objects in an image) [85]. For that, the recognition accuracy for the second network was higher than for the first network (a difference of a few/several percent). Thus, the choice of an appropriate model is a trade-off between the accuracy and speed of detection. For the above reasons, both types of neural network are often used [86]. Since a lot of work has been carried out worldwide in recent years in terms of using and improving the effectiveness of multiple DNN architectures, one can expect continuous improvements in both the recognition accuracy and speed of flame and smoke detection. Neural networks make it possible to detect flames not only in forests or wilderness areas, but also in enclosed facilities. Their application also extends to places where, for example, due to high temperatures or dust, the use of classical sensors is severely hampered (e.g., foundries, sand dryers, heat treatment plants, etc.).

On the other hand, a future solution may be the use of acoustic technology not only in firefighting actions, but also in fire detection. This is due to the fact that sound is generated by the heat flow associated with the flame (in practice, many materials that are exposed to heat cause acoustic emissions, which includes their heating, pyrolysis, and combustion process) [87]. These emissions take on a variety of forms, from crackling or popping to the release of gases. This is important because acoustic sensors can detect flames before a difficult-to-extinguish fire breaks out. Levterov presents aspects of the use of acoustic waves in the field of preventive measures, including the detection of flames in the initial stage of fires using spectral signal processing techniques, e.g., [88,89]. Moreover, in the long term, acoustic technology may also find application in the fight against pollution that is the result of fires. 

**Figure 1:**
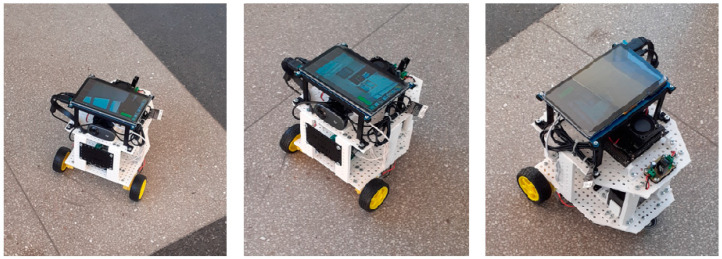
An intelligent (optional) component for the fire extinguisher to detect flames and smoke.

Through the use of an acoustic extinguisher electrically connected to an AI (Artificial Intelligence) module, it becomes possible to automatically detect the source of flames from a particular type of organic substance and, in the case of positive detection, immediately generate the acoustic waves necessary to extinguish them. Images from both visible and infrared bands may be applied for flame detection (in the second case, higher pixel intensities are observed [90]). For practical implementation, a number of techniques and libraries can be used, including Matplotlib, OpenCV, TensorFlow, NumPy, and Imutil, among others. An example of a flame and smoke detection system dedicated to acoustic technology is provided in Figure 1. It can be a module permanently implemented in a fire extinguisher or, as shown in Figure 1, a standalone platform that allows the remote detection of flame outbreaks. More information on this subject can be found in the publications: [84,85,86,91,92]. 

Whether the optional smart platform is permanently installed in the acoustic extinguisher or is an additional mobile (portable) component of the acoustic extinguisher, the recognition results will be the same and depend on the effectiveness of the deep neural networks to detect flames and smoke. The step-by-step algorithm is described in the articles of Ivanov et al. [84,85,86,91,92]. Such a robotic platform makes it possible to test various flame detection algorithms and assess the source of the flames, which is important for the growing use of robots [93,94]. The installation of a physically connected driving device to the extinguisher may allow the extinguisher to move and change direction, so that the outlet of the device is always directed towards the source of the flames and is kept at the correct distance from the fire source using ultrasonic and temperature sensors [85]. For this purpose, an algorithmic method may be applied to design control systems and structural implementations of systems can be implemented, e.g., [95,96]. In practice, concentrating the acoustic beam makes it possible to improve the range of the extinguisher and reduce the required acoustic power [97,98]. However, despite the many advantages, the use of the acoustic method is associated with certain limitations (including design constraints, the size of the extinguisher, and the limited range of the extinguisher—depending, among other things, on the power delivered to the sound source, which determines the difficulties when extinguishing large fires). Therefore, more research is needed to determine the limits of the use of this technology. A separate issue is the continuation of research focused on the safety of living beings (including humans) when exposed to acoustic waves, as well as research in terms of the strength of materials exposed to low-frequency acoustic waves [83]. To increase the scalability of flame extinguishing by acoustic waves, acoustic systems consisting of multiple sound sources may be necessary for larger fires, allowing effective flames in an improved acoustic field [91]. Methods for detection and, in particular, acoustic flame extinguishing can have great potential for future applications, as exemplified in industrial plants, production halls, organic material warehouses, and even (due to specificity) in the space segment.

Another important point that requires detailed research is the effect of organic compound combustion products on the acoustic parameters of devices used for monitoring in a smoke-filled area. Combustion products significantly change the acoustic properties of the environment. This affects the propagation speed of sound waves, their amplitude, and attenuation. In particular, a high concentration of suspended particles resulting from combustion can lead to an increase in the wave resistance of the medium, which changes the acoustic impedance and creates difficulties for the effective transmission of signals. The lack of the sufficient study of these aspects complicates the development and implementation of effective acoustic monitoring systems under conditions of smoke and dust, which are often observed during emergency and rescue operations.

Given that today a large part of environmental objects are organic compounds, often of a polymeric nature, the issue of investigating the impact of these compounds on the possibility of monitoring in a smoke-filled area is also relevant. For data transmission from hard-to-reach locations, modern data capture techniques and wireless communications can be used, taking into account propagation factors [99,100,101,102,103].

In addition, temperature gradients that occur during fires and other emergency situations also have a significant impact on the acoustic characteristics of the environment. An increase in temperature leads to an increase in sound speed, which can negatively affect the accuracy of measurements and the overall effectiveness of monitoring. As was shown in studies [104,105], the sound dependence of the sound speed on the temperature gradient is linear, but in conditions of high temperatures, more complex nonlinear effects may occur, which require further study.

On the other hand, acoustic instruments used for monitoring in such conditions often face additional difficulties related to changes in the acoustic impedance of the environment due to the high concentration of suspended particles. This leads to the significant absorption of acoustic waves and a decrease in the efficiency of their propagation. Modern research indicates the need to develop new approaches to increase the reliability of acoustic systems in environments with a high concentration of combustion products [106,107].

The lack on systematic studies of the impact of the combustion products of organic compounds on the acoustic parameters of devices is a significant scientific and technical problem that holds back the development of acoustic technologies for use in environments with a high concentration of suspended particles, elevated temperatures, and other factors affecting the propagation of acoustic waves. A comprehensive study of the influence of these factors will allow the development of new methods to increase the accuracy, reliability, and efficiency of acoustic systems used for orientation and information transmission in smoke-filled areas and will contribute to increasing the level of security of premises and people, including specialists of special services.

Thus, this purpose of the work is to investigate the possibilities of the acoustic method for extinguishing organic compounds, including monitoring in a closed three-dimensional space with conditions of dense smoke due to the burning of organic materials (including polymer), dust, and elevated temperatures.

This paper experimentally investigates the possibilities of the acoustic method in fire extinguishing, further developing the ideas about the possibilities of this method using the combustion of various polymers, which is the novelty of the work. Using the example of extinguishing a paraffin wax candle, correlation dependencies between the parameters of extinguishing and the distance to the ignition source are determined, and the effect of combustion of various polymers on the acoustic parameters of the environment and the possibility of using them to monitor this environment are also shown, which is a unique contribution of this work.

## 2. Materials and Methods

The research part of this paper is typically experimental in nature. In particular, the literature review method and the comparative method were used to supplement the content with the most important information on the subject of the article (state of the art). Elements of system analysis appeared in the theoretical part, which made it possible to present a clear structure of the analyzed topic. The results of the experimental attempts were analyzed using the comparative method. Analytical and individual case methods were also applied for this purpose. A model experiment was also used to study the effect of combustion products on the propagation of acoustic waves, a full-scale experiment was carried out to check the functioning of acoustic systems in real conditions, and the statistical processing of the experimental data was carried out to identify regularities and dependencies.

The work involved burning a number of organic substances: paraffin wax, wood, paper, plastic, polystyrene, textiles, gypsum plasterboard, and engine (motor) oils. 

The characteristics of some of these substances have already been given above. Paraffin wax is a mixture of hydrocarbons, consisting of hydrocarbons with a straight chain, having a carbon number mainly greater than C20 [108]. Gypsum plasterboard consists of a gypsum core (calcium sulfate dihydrate), which is sandwiched between two layers of paper and has high fire-resistant properties [109,110]. Engine (motor) oils are a mixture of petroleum-based hydrocarbons, polyalphaolefins, and some other hydrocarbons [111].

The first part of the research involved the use of the acoustic method in the range of two carrier frequencies of the extinguisher, i.e., 15 and 19 Hz, depending on a distance of the flame source from the outlet of the waveguide, which is also the outlet of the extinguisher. These frequencies were chosen to obtain a small impedance (11.4 Ω). Its selection translates into an acceptable level of the vibration of the diaphragm of the sound source and its effectiveness, i.e., optimal acoustic energy transfer. Therefore, these frequencies oscillate around 17 Hz (the upper value plus and the lower value minus 2 Hz). Their selection was preceded by the determination of the transmission characteristics of the acoustic system. The selection of the methods was evaluated in terms of their ability to achieve specific research goals.

For the purpose of the experiment, a point source of the flame was used, i.e., a candle with a flame height of about 2 cm, whose distance from the outlet of the extinguisher was varied from 20 cm to 70 or 80 cm (with a step of 20 or 10 cm in the case of the last distance), so it was possible to determine unambiguously whether the flames were successfully extinguished (this is important because when a diffuse fire source was used, they sometimes returned). The organic substance that was extinguished was paraffin wax in these attempts.

**Figure 2:**
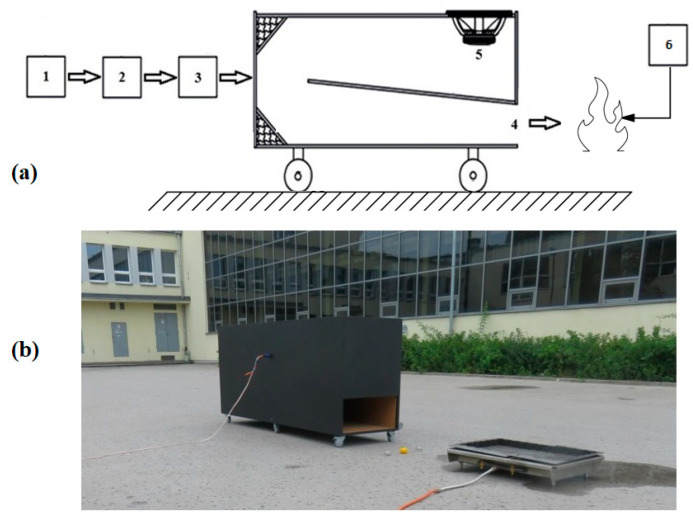
(**a**) Elements of the acoustic extinguisher (illustrative view): (1) generator, (2) modulator, (3) amplifier, (4) waveguide outlet, (5) sound source, and (6) flame source (i.e., candle, mock-up, etc.) exposed to acoustic waves; (**b**) actual acoustic extinguisher.

A laboratory stand (Figure 2) was built for experimental attempts to extinguish flames using acoustic waves, with an acoustic extinguisher as the overriding component. It generates acoustic waves until the flames are completely extinguished. The test stand is universal (it allows us to change the parameters of the acoustic wave, in particular, its frequency, as well as its shape and modulation, as needed). Using a laboratory stand, it is possible to test the possibility of extinguishing flames originating from various materials with acoustic waves (i.e., paraffin wax, flammable gases, etc.). The tests were carried out outdoors (in windless conditions) on the same day with a background sound level oscillating around 65 dB. During these attempts, the maximum power delivered to the acoustic extinguisher did not exceed 1000 W. In practice, the power level required to extinguish the flames and the sound pressure level at the point where the flames were extinguished were investigated. For this purpose, a Class 1 SVAN 979 meter (SVANTEK Ltd., Warsaw, Poland) was used for measurements according to IEC (International Electrotechnical Commission) 62672-1:2013. The acoustic measurement time for each attempt was equal to 5 s.

The acoustic extinguisher shown in Figure 2, in addition to the waveguide, consists of a power supply (portable battery or mains), a Rigol DG4102 generator (Rigol Technologies, Warsaw, Poland) with a modulation function, a Proel HPX2800 power amplifier (Proel s.p.a., Sant’Omero, Italy) which is electrically connected to the waveform generator, and a sound source B&C 21DS115 (B&C Speakers s.p.a., Bagno a Ripoli, Italy). A nominal power of the sound source was equal to 1 700 W (one thousand seven hundred watts). The amplifier input was supplied with a voltage of 1 V RMS. The role of the generator (usually with a modulation function) is to generate a suitable low-frequency waveform, for which the signal is provided to the power amplifier. The sound source, in turn, converts the signal so that there is an acoustic field generated by emitting waves with the energy necessary to extinguish the flames (without causing unwanted effects in the extinguished areas, hence the technique is called non-invasive) [112]. The tip of the waveguide is directed toward the flames. Fire extinguishers can use additional mechanisms in their operation that affect the process of extinguishing flames (e.g., connection ports, additional energy transducers, baffles, barriers, and elements that reflect waves, elements that store the energy of acoustic waves due to additive interference, attachments for the precise direction of acoustic waves, caps that increase the acoustic pressure at the outlet of the waveguide, etc.) [113]. This is important because, in particular, the close environment may affect the effectiveness of flame extinguishing with acoustic waves [114]. Generated, amplified, and focused sound waves of low frequency and high or very high acoustic power allow the creation of local conditions in which the flame is unable to sustain itself, as its continuous exposure in the acoustic field leads to its complete extinguishment without the need for chemicals [39].

Practical considerations have influenced the extinguisher to select a one-sided closed waveguide with a length of 4.28 m, assuming a sound propagation speed of 343 m/s. A notable feature is that the rectangular cross-section of the waveguide narrows towards the outlet of the acoustic extinguisher. With the reduction in the outlet, a favorable effect of increasing the sound pressure level at the outlet of the waveguide was observed, taking into account the physical properties of sound propagation. In the other case (open version), its length would have to be doubled, which for acoustic waves of such a low frequency would be difficult to achieve for practical reasons. Therefore, the extinguisher has a one-sided closed waveguide (acoustic waves are amplified due to the phenomenon of acoustic resonance), which is used in the acoustic technique of flame extinguishing.

**Figure 3:**
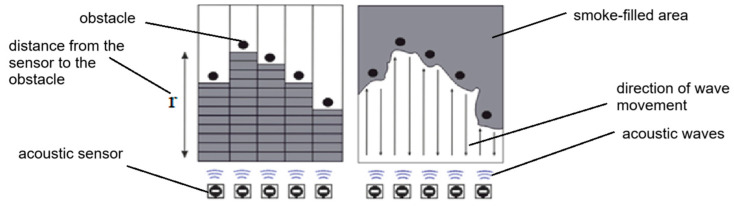
Scheme of operation of the acoustic device.

The second part of the study, aimed at investigating the features of monitoring in a smoky room, is based on the application of an acoustic device, the principle of which is based on the use of echolocation. This approach allows for the more effective detection of objects and obstacles, which improves the ability of specialists to quickly and accurately assess the surrounding situation. The device, thanks to the analysis of reflected acoustic waves, makes it possible to detect obstacles in a space, which allows to ensure an effective level of monitoring in a smoke-filled area during the combustion of organic substances. Figure 3 shows a graphic representation of the functioning of the device.

**Figure 4:**
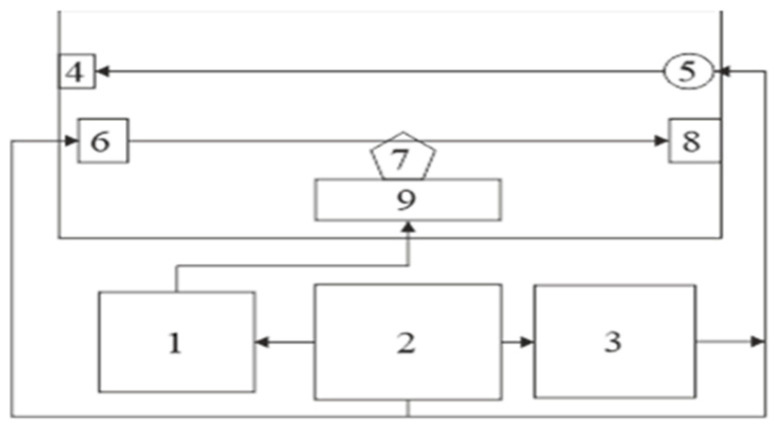
Functional scheme of the experimental setup for studying the influence of combustion products on the propagation of acoustic waves.

The functional scheme of the device (Figure 4) consists of a closed environment model and its components: 1—smoke control unit; 2—power supply unit; 3—control unit; 4—optical sensor; 5—heating element; 6—acoustic detector; 7—a sample of the researched material; and 8—acoustic sensor.

**Figure 5:**
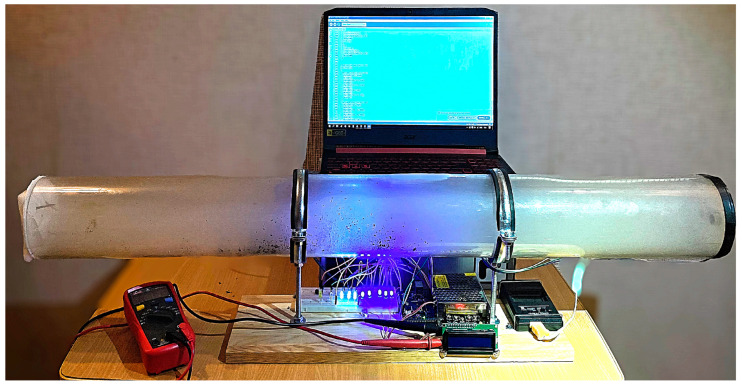
General view of a laboratory setup for studying the influence of combustion products on the propagation of acoustic waves.

**Figure 6:**
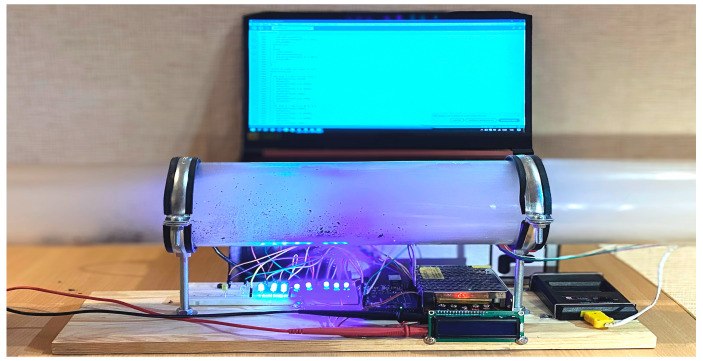
Detailed view of the display system and power supply units of the laboratory installation for studying the effect of combustion products on the propagation of acoustic waves.

To solve the problems, field studies were carried out with the help of an experimental setup (Figure 5 and Figure 6). The glass flask (Figure 5 and Figure 6) has a length of 1 m, a diameter of 10 cm, and a volume of 7.85 L. 

At the beginning of the experiment, the flask is completely transparent; that is, the optical permeability of the medium is close to 100%. The light sensor captures the signal from the LED without interference. A sample of organic material is set on fire, and the process of releasing combustion products (suspended particles and gases) begins. The particles gradually fill the flask. In the process of combustion, the products fill the flask, reducing the optical permeability of the medium. A sensor placed on the opposite side of the flask gradually registers a decrease in the intensity of light from the LED. At the moment when combustion products reach a certain (limit) concentration, the sensor stops registering the signal from the LED. This indicates that the optical permeability of the medium has reached a minimum (close to 0%). Time fixation is carried out from the moment of the beginning of the release of the combustion products until the complete loss of the optical transparency of the medium in the flask.

The experiments were repeated within stable parameters five times for each set of conditions of the medium (temperature of medium, acoustic wave incidence angle, concentration of suspended particles, and acoustic impedance) to ensure the stability and reproducibility of the results. The error of determinations obtained using standard statistical approaches did not exceed 5%.

## 3. Results and Discussion

### 3.1. Study of the Possibilities of the Acoustic Method for Detecting and Extinguishing Fires of Organic Substances

The acoustic technique is currently in the testing phase. Since, as previously demonstrated by the co-authors of this paper (bibliography), both unmodulated and modulated waves can be applied to extinguishing, this paper presents results on the possibility of extinguishing flames using sinusoidal waveforms amplitude-modulated (AM) by a square waveform (M_Freq_ = 0.125 Hz). The posted results experimentally confirmed the validity of the theory that such waves can be used successfully to extinguish flames. The results of the research and development work presented in this article are related to experimental attempts to extinguish flames using a prototype acoustic extinguisher with high and very high acoustic power, created based on the structural concept developed (the result is a product—creation) within the framework of the competition co-financed by the Ministry of Science and Higher Education of the program “Innovation Incubator +” [112]. The tangible goal of the research within this program is the presentation of the results from actual attempts of extinguishing, including organic substances, as well as the explanation and description of the principle of the acoustic method, taking into account the physical apparatus of acoustics, considering the molecular point of view and the propagation of acoustic waves that effectively extinguish flames. This approach to the subject made it possible, from a conceptual point of view, to explain the phenomenon in a practical way and to use acoustic waves to control and extinguish flames (the purpose of the extinguisher is to extinguish flames from various substances). For this reason, scientific efforts within the framework of the “Innovation Incubator +” (InIn+) project have focused, inter alia, on considering the physical properties of propagated acoustic waves (to control the combustion process and the extinguishing of flames), the analysis of convection disturbances, and the study of the extinguishing properties of unmodulated and modulated waves. In particular, these analyses can be applied to detail the phenomena accompanying the propagation of acoustic waves in confined or enclosed spaces. 

Acoustic technology is effective in extinguishing organic flames. The reason why the acoustic method is in the center of interest is the many benefits of its application in fire protection, including its versatility, the possibility of multiple applications, and the adjustment of the extinguisher (waveform and modulation) to a particular flame source [82,83,84]. These factors significantly reduce operating costs and simplify the maintenance of the acoustic fire protection system, which, in addition to the portable version, can be permanently installed, inter alia, on the foundations of a building (such as an industrial plant).

As mentioned previously, this paper presents the results on the extinguishing of flames using the acoustic method over a range of two extinguisher operating frequencies, i.e., 15 and 19 Hz, depending on a distance of the flame source from the extinguisher outlet. For the experiments, a point source of flame was used, i.e., a candle filled with a paraffin wax with a flame height of about 2 cm, whose distance from the extinguisher outlet was changed from 20 cm to 70 or 80 cm (with a step of 20 or 10 cm in the case of the last distance). Using a point source of flames, as opposed to diffuse sources, it was possible to clearly determine whether the flames were successfully extinguished. The power level required to extinguish the flames and the sound pressure level at the point where the flames were extinguished were examined. As previously indicated, all measurements were made under similar conditions with the background sound level of about 65 dB. It should be noted that in an environment with high noise levels, acoustic waves, depending on the frequency used, can be more or less susceptible to ambient sounds (generally in the range of about 15 to 60 Hz). For this paper, the results of the experimental attempts on the possibility of extinguishing flames using modulated acoustic waves are presented (this technique has not yet been fully discovered, so there was a need to fill the literature gap in this area). In the experimental part, amplitude modulation (AM) was applied—a sinusoidal waveform modulated by a rectangular waveform (M_Freq_ = 0.125 Hz). These experimental results have potential application in the testing of the acoustic extinguisher with a high and very high acoustic power and in clarifying the principle of its operation.

**Figure 7:**
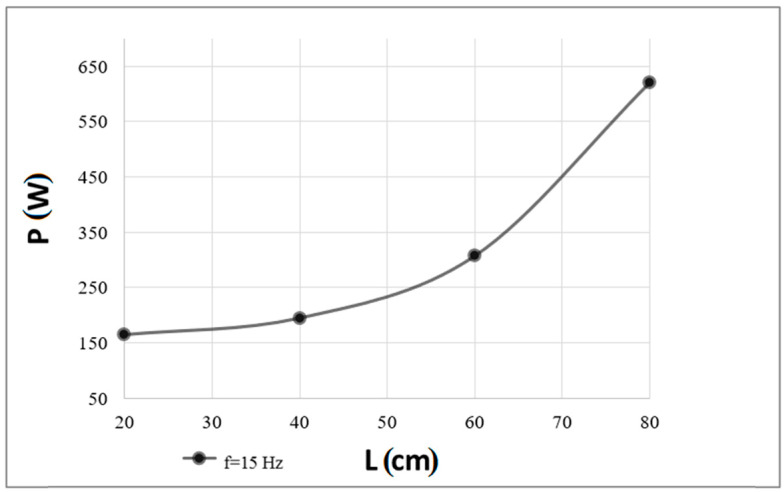
Minimum electrical power (P) that must be delivered to the extinguisher to extinguish the flames depending on a distance (L) from the fire extinguisher outlet (a case of the carrier wave of 15 Hz).

Below are presented the results for the necessary electrical power that had to be delivered to the acoustic extinguisher to extinguish the flames and the recorded sound pressure level at the point where the flames were successfully extinguished, as a function of a distance from the waveguide outlet (20, 40, 60, and 70 or 80 cm), for the carrier wave frequencies analyzed (15 and 19 Hz). Figure 7 shows the results for the required power to extinguish the flames at 15 Hz as a function of distance from the device outlet.

**Figure 8:**
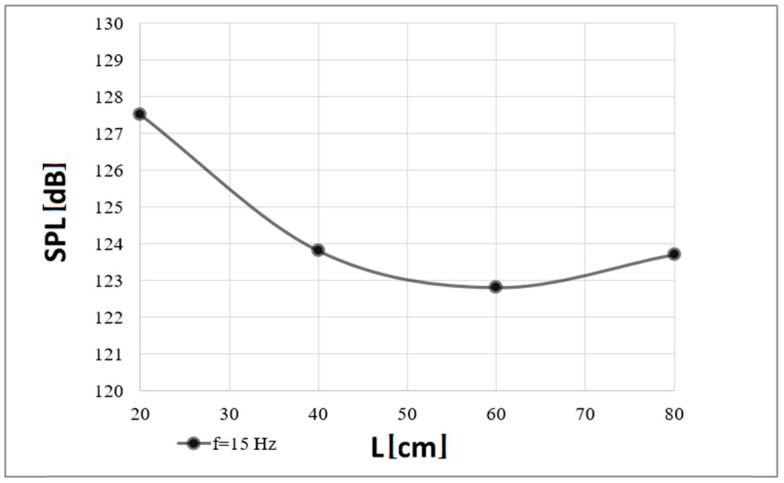
Minimal Sound Pressure Level (SPL) at which complete extinguishment of flames was recorded depending on a distance (L) from the fire extinguisher outlet (a case of the carrier wave of 15 Hz).

The minimum sound pressure level at which the flames were completely extinguished as a function of a distance from the device outlet is presented in Figure 8.

**Figure 9:**
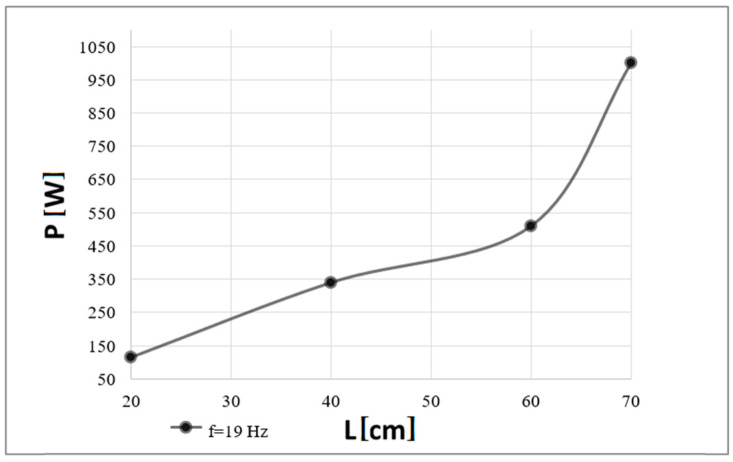
Minimum electrical power (P) that must be delivered to the extinguisher to extinguish the flames depending on a distance (L) from the fire extinguisher outlet (a case of the carrier wave of 19 Hz).

**Figure 10:**
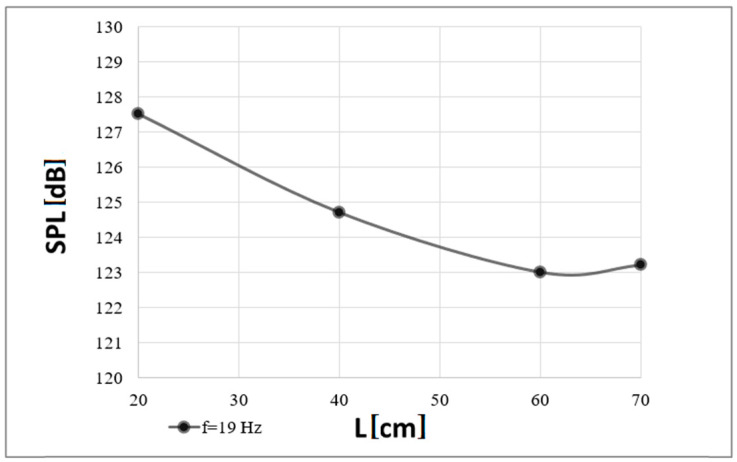
Minimal Sound Pressure Level (SPL) at which complete extinguishment of flames was recorded depending on a distance (L) from the fire extinguisher outlet (a case of the carrier wave of 19 Hz).

Correspondingly, Figure 9 and Figure 10 present the analogous measurement results obtained for the higher frequency of the acoustic wave analyzed, i.e., 19 Hz.

The results of the sound pressure level obtained for the second of the acoustic wave frequencies analyzed, i.e., 19 Hz, seem interesting against this background (Figure 10).

The conducted research shows that the extinguishing effect depends on a frequency. A correlation can be observed between an increase in power and an increase in frequency, with differences due to the operating point of the acoustic extinguisher. Taking into account the identification of the fire extinguisher parameters, the closer to the frequency at which the minimum impedance was recorded, the lower power required to extinguish the flames. For both frequencies analyzed, as the distance between the waveguide outlet and the flame source gradually increased (with a step of 20 or 10 cm in the case of the last distance), more and more electrical power had to be delivered to the sound source to observe the phenomenon of the complete extinguishment of the flames (this regularity applied to all analyzed distances from the flame source). The recorded necessary powers to extinguish the flames were generally higher for the second of the analyzed frequencies, i.e., 19 Hz (except for the data obtained for the smallest of the distances at which similar results were noted). By comparing these two frequencies, for a distance of the flame source from the extinguisher outlet equal to 20 cm, a power difference of 50 W was recorded, for a distance equal to 40 cm—145 W, for a distance equal to 60 cm—203 W, and for the last distance—380 W (however, it should be noted that for the 19 Hz frequency, the maximum distance was 70 and not 80 cm due to the safety of the woofer). The implication of this is that the deviation from the resonant frequency affects the increase in the electrical power required to extinguish the flames, which for a distance of 70 cm exceeded 1 000 W. Therefore, for the frequency of 19 Hz, the experimental results are limited to distances up to 70 cm.

The sound pressure level at which complete extinguishment of the flames occurred for both frequencies was similar (it was in the 120–130 dB range), with a greater spread of its values recorded for the first of the analyzed frequencies, i.e., 15 Hz. For the smallest distance, i.e., 20 cm, the sound pressure levels obtained were the same. For distances of 40 and 60 cm, differences of less than 1 dB (0.9 and 0.2 dB, respectively) were recorded. Comparable results were also obtained for the last distances analyzed between the flame source and the waveguide outlet, i.e., 80 and 70 cm, respectively.

An analysis of the experimental attempts allows one to note a relationship between the measured sound pressure levels and the distance from the waveguide outlet. An inversely proportional decrease in the sound pressure values is observed with an increase in the distance of the flame source from the waveguide outlet. Since the diagrams (Figure 3, Figure 4, Figure 5 and Figure 6) were obtained under the same environmental conditions, the shapes of the characteristics are similar. 

The presented results have experimentally confirmed the validity of the theory that acoustic waves can be successfully applied to extinguish flames. In principle, low-frequency acoustic waves are particularly useful and effective in extinguishing flames due to the significant turbulence they cause in the flame. The benefit then is the low power that must be delivered to the sound source to observe the complete extinguishment of the flames. On the other hand, their use is associated with the significant vibration of the diaphragm of the sound source due to the mismatch of waveguide and loudspeaker conditions. At slightly higher frequencies, the better focusing of the propagated acoustic beam is observed. 

In practice, as shown in Figure 7 and Figure 9, the acoustic extinguisher requires the delivery of more power than the thermal power obtained by burning the substance. Therefore, taking into account energy issues and power consumption, for a sound source, such as a woofer (as an electroacoustic transducer) that converts electric current into an acoustic wave, the efficiency is low (on the order of a few percent). For this reason, in the future, the acoustic method assumes the search for other, more efficient and sophisticated, sound sources than the speaker. It should also be noted that during the experiments performed, much lower powers than the nominal power of the loudspeaker were applied, with successful extinguishing attempts. Therefore, using higher powers, it will become possible to obtain an energy reserve to effectively extinguish the flames in the event of an unfavorable change in conditions at the flame extinguishing site. Taking into account the advantages of using this technique in relation to other traditional methods of fire protection, including its versatility of use, environmental friendliness (acoustic waves are not a chemical creation), the unlimited operation time of the extinguisher being dependent only on the supply of electricity to it, the absence of the cyclic pressure tests of the tank, the absence of a human presence in the place of extinguishing, and many others, it is assumed that this method can be used especially to extinguish firebreaks, effectively preventing the spread of fire.

### 3.2. Justification of the Effect of the Smoke-Filled Rooms Due to the Burning of Various Organic Compounds on Its Acoustic Characteristics

#### 3.2.1. Determination of the Acoustic Characteristics of the Smoke-Filled Environment Due to the Combustion of Organic Substances

The increased temperature of the environment, characteristic of fires, significantly affects the speed of the propagation of acoustic waves, changing their physical parameters and making it difficult to predict propagation trajectories. When critical temperatures are reached during a fire, the acoustic properties of not only air, but also other materials, such as walls and ceilings, change. This leads to significant changes in the wave resistance indicators of the environment, which complicates the monitoring process in conditions of the smoke-filled areas due to the burning of organic compounds. In this case, acoustic waves are one of the key means of transmitting information in a smoke-filled area, when traditional methods of communication can be disrupted due to the presence of physical obstacles or adverse conditions. This is especially true for emergency situations, such as man-made disasters or armed conflicts, where the physical parameters of the environment greatly complicate the propagation of signals. The improvement of information transmission technologies using acoustic waves, taking into account specific parameters of the environment, such as the concentration of suspended particles (formed during the combustion of organic compounds), temperature, and wave resistance, is a necessary condition for ensuring monitoring under complex and heterogeneous conditions.

As mentioned above, the different chemical composition of compounds formed during the combustion of various organic substances (for example, the presence of methane and soot, formed due to incomplete combustion), causes the additional scattering of acoustic waves.

When the possibilities of monitoring in smoke-filled areas during the combustion of organic compounds are investigated, the limits of the concentration of suspended particles are determined on the time required to fill the flask to the state when the optical transparency is reduced to a minimum. The results of the research are presented in Table 2.

According to the results obtained, the products of the combustion of the materials show a different degree of influence on the propagation of acoustic waves, which is determined both by the velocity of reaching the critical concentration of the combustion products and by the change in the physical parameters of the environment. In particular, motor oil and drywall stand out as extreme examples in terms of exposure.

Motor oil exhibits the greatest influence on acoustic waves. In 15.7 s, the critical concentration of combustion products is reached, and the temperature coefficient (K = 1.04) indicates significant changes in the characteristics of the medium. The 25% loss of velocity is explained by the presence of heavy aerosols and toxic substances formed during combustion. This makes grease one of the most unfavorable materials for the propagation of acoustic waves, creating significant obstacles to the operation of acoustic navigation systems.

Gypsum plasterboard can be characterized as a relatively favorable material from the point of view of the propagation of acoustic waves, since its combustion products have a minimal effect on the acoustic properties of the environment. The time to reach the critical concentration is 78.5 s, which is the longest indicator. The temperature coefficient (*K* = 1.018) and the 8% loss of velocity testify to the limited influence of its combustion products on the physical properties of the medium. In the presence of gypsum plasterboard combustion products, acoustic waves retain their characteristics.

Based on research in work [115], the loss of velocity (Δ*ν*) of acoustic waves during the passage of acoustic waves is determined according to Formula (7):

(7) $$\Delta \nu =\frac{\nu_{in}-\nu_{fin}}{\nu_{in}}\cdot 100\%$$

where *ν_in_* is the velocity of sound before the release of combustion products, and *ν_fin_* is the velocity of sound after filling the glass flask with combustion products until reaching the limit concentration level.

Specialized equations are used to determine the coefficients of the angle of incidence, absorption, temperature, and wave resistance, which take into account the influence of environmental conditions on the propagation of acoustic waves. They describe the relationship between the physical parameters of the environment and the acoustic characteristics, which then allows for the accurate adjustment of the indicators of acoustic devices.

**Figure 11:**
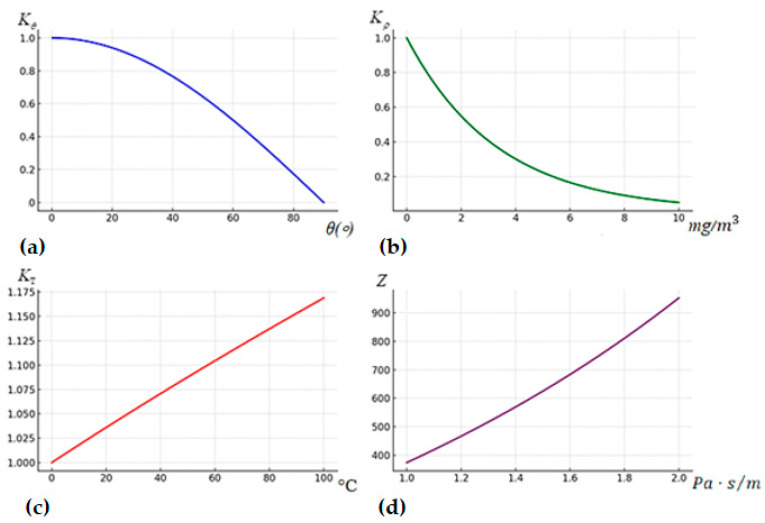
Graphic display of the dependence of the studied coefficients: (**a**) coefficient for the angle of incidence (*K_θ_*); (**b**) absorption coefficient (*K_ρ_*); (**c**) temperature coefficient (*K_T_*); and (**d**) coefficient of acoustic impedance (*Z*) on the parameters of the environment with combustion products.

For each of the coefficients, a graphical dependence (Figure 11) was developed to provide a deep understanding of the influence of the relevant factors on the propagation of acoustic waves.

The key coefficients that affect the propagation of acoustic waves are presented in Figure 11 and described below:

(a) Acoustic waves at different angles of incidence are reflected or absorbed by surfaces with different intensities. According to [116], the correction coefficient for the angle of incidence (*K_θ_*) can be determined through the following relationship (8):

(8) $$K_{\theta }=\cos(\theta )$$

where θ is the angle of incidence of the wave on the obstacle, °. As the angle of incidence of the wave on the boundary of the media increases, the value of the coefficient decreases, which indicates a decrease in the efficiency of the energy transfer. At large angles of incidence, most of the wave energy is reflected from the surface, which limits the penetration of the wave into another medium and leads to greater energy losses.

(b) The concentration of suspended particles affects the coefficient of absorption and the scattering of acoustic waves (absorption coefficient, *K_ρ_*). When the concentration of particles (for example, dust or combustion products) and the environment increases, the intensity of the propagation of the acoustic wave decreases, despite the increase in the number of points of interaction between the wave and particles. The effect is due to several key factors. When an acoustic wave collides with particles, part of its energy is dispersed in different directions. This reduces the amplitude of the wave in the direction of its original propagation. The energy of the acoustic wave is converted into heat or other forms of energy. Also, with a high concentration of particles in the medium, interference processes occur, in which the superimposition of waves in antiphase leads to a partial extinction of energy. This leads to an exponential decrease in the intensity of the sound signal with distance.

Therefore, the absorption coefficient of acoustic waves by the concentration of suspended particles is proportional to the concentration of suspended particles and is determined according to [117] by Formula (9):

(9) $$K_{\rho }=e^{-\alpha \cdot d}$$

where α is the absorption coefficient of waves in the medium (depends on the type of particles) and *d* is the distance that the wave travels through the medium with suspended particles, m.

(c) In the initial section of the graph, with the increasing temperature, the value of the temperature coefficient increases rapidly, showing an almost linear dependence. This indicates that the temperature significantly affects the medium, reducing its density and increasing its elasticity, which contributes to the increase in the speed of the propagation of acoustic waves. However, with an additional increase in temperature, the growth rate gradually slows down and reaches a stable level. This is explained by the fact that after reaching a certain temperature, changes in the density of the medium become insignificant, and the effect on the speed of sound waves is less pronounced. Thus, although at the initial stage the effect of a temperature increase is significant and close to linear, at high temperatures it gradually weakens due to the saturation of the physical properties of the medium. 

The temperature coefficient indicates that with an increase in temperature, the velocity of the propagation of acoustic waves in the medium increases, which confirms the expression (10) according to the studies [118]:

(10) $$K_{Τ}=\frac{\nu }{\nu_{0}}=\sqrt{1+\frac{T}{T_{0}}}$$

where *ν*_0_ is the velocity of the propagation of acoustic waves at room temperature, m/s; *ν* is the velocity of propagation of acoustic waves at the current temperature of the medium, m/s; *T*_0_ is the room temperature, 293 K; and *T* is the current temperature of the medium, K.

(d) The coefficient of acoustic impedance (*Z*) determines the efficiency of the sound wave transmission between media and is calculated by Formula (11), according to the work [119]:

(11) $$Z=\frac{Z_{2}-Z_{1}}{Z_{2}+Z_{1}}$$

where *Z*_1_ and *Z*_2_ are the acoustic impedances of the media through which the wave passes. 

The coefficient of acoustic impedance shows how much of the sound wave energy will be reflected or transmitted across the boundary of the medium. The greater the impedance difference, the more energy is reflected, reducing the efficiency of wave transmission.

Acoustic impedance (measured in Pa·s/m) determines how much the medium resists the propagation of acoustic waves. An increase in impedance values leads to the greater reflection of sound waves at the interface between media and reduces the energy transfer across the interface. The dependence is important for understanding how acoustic waves interact with the boundaries of materials with different properties.

The general equation to describe the interaction of acoustic waves with the environment, taking into account the coefficients accordingly, will have the form (12):

(12) $$\nu (\theta ,\rho ,Τ,Z)=\nu_{0}\cdot {Κ}_{T}\cdot {Κ}_{\theta }\cdot e^{-\alpha \cdot d}\frac{1}{\sqrt{Z}}$$

The transformation of physical parameters, such as the temperature of the environment, the angle of incidence of the wave, the concentration of suspended particles, and the acoustic impedance, into the corresponding coefficients—the temperature, angular correction, absorption, and acoustic impedance—ensures the standardization of data and the simplification of mathematical models. The proposed general formula for calculating the propagation speed of acoustic waves takes into account the errors of the specified parameters of the environment. It is shown that changes in the parameters of the environment (such as the temperature, particle concentration, wave incidence angle, and acoustic impedance) affect the corresponding coefficients: the temperature coefficient, absorption coefficient, angular correction, and acoustic impedance coefficient. These coefficients are intermediate indicators that characterize the behavior of sound waves under variable conditions.

The value of the coefficients and the corresponding physical laws will allow for determining the influence of each parameter on the propagation of acoustic waves in environments with combustion products. This approach makes it possible to clearly link the physical parameters of the environment with the characteristics of acoustic waves, which is important to model acoustic processes and predict signal transmission conditions under aggressive environmental conditions.

#### 3.2.2. Experimental Determination of the Influence of the Temperature of Combustion Products on the Physical Properties of Acoustic Waves

The study [120] carried out an analysis that showed that each substance has its own acoustic spectrum during combustion, since its physical and chemical properties determine a specific frequency response. This spectrum is formed as a result of the emission of acoustic waves during the chemical reactions of combustion or pyrolysis. Depending on the type of material (cellulose, plastic, metals, etc.), these characteristics can be significantly different, which allows one to identify the substance by its acoustic properties and to investigate the effect of the temperature of the combustion products on the propagation of acoustic waves.

Different combustible materials are characterized by different temperatures of combustion products, which is due to their chemical composition, heat of combustion, and the amount of oxygen involved in the combustion process. For example, the combustion temperature of cellulosic materials differs from the temperature at which plastic or metal burns. These temperature differences affect the acoustic characteristics of the environment, in particular, the speed of sound, its absorption, and reflection, which must be taken into account when analyzing the propagation of acoustic waves.

Due to the fact that the speed, force, direction of the flow (effect of the temperature gradient), as well as the temperature are constantly changing in space, the propagation of sound waves occurs constantly under new conditions and the attenuation of sound increases due to the reflection, scattering, and lengthening of the path, which passes through sound (at a temperature of −20 °C, sound travelling at 318 m/s, and at a temperature of +20 °C—344 m/s).

According to the formula of Navier Laplace [121], which describes the dependence of the velocity of the propagation of acoustic waves on the temperature of the medium, the expression for the velocity of sound has the form (13):

(13) $$\nu =\sqrt{\frac{\chi R_{0}}{\mu }}T$$

where $\chi =\frac{c_{p}}{c_{v}}$ is the ratio of heat capacities at a constant pressure and constant volume, μ is the molecular weight of the gas, kg/mol; *R*_0_ is the molecular weight of the gas, (8.314∙J)/((mol∙K)); and T is the temperature, K. 

The influence of the temperature of the products of the combustion of the materials on the readings of the acoustic device was measured. The results of the experiment are shown in Table 3.

**Figure 12:**
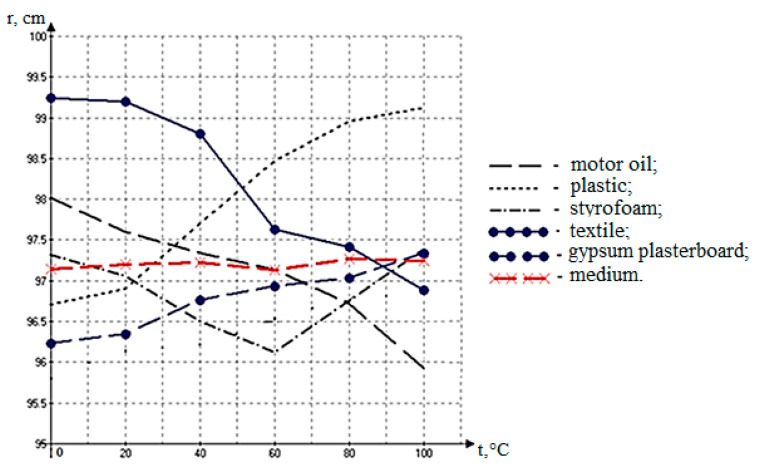
Graph of dependence of the temperature of the products of combustion of materials on the readings of the acoustic device.

Once the data had been removed, a graphical display was created (Figure 12), which allows you to change the value of the acoustic sensor before it changes at different temperatures of the monitored material.

Based on the results of the expansion, the average value of the adjusting parameter of the acoustic device (r_Δavrg_) = 0.028 m at a distance of 1.0 m is indicated. 

An analysis of the graph (Figure 12) shows that with the increasing temperatures of the combustion materials, the fluidity of the acoustic waves and their polishing changes significantly. It is important to note the importance of maintaining the temperature gradient in the structures of acoustic devices for the precise alignment of the stage before the transient. The results confirm that materials with different temperature readings of combustion products have a different impact on the characteristics of acoustics, which also affects the accuracy of the readings of the device.

## 4. Prospects for Applying the Approach

In the future, further attempts using the acoustic extinguisher for other waveforms, modulation techniques, and frequencies are planned, so that the possibilities of extinguishing flames using acoustic technology can be explored as well as possible, thus filling the literature gap in this area. The results obtained for flame-retardant materials and various acoustic wave parameters may also be interesting [122,123,124,125,126,127,128,129,130].

Another further direction of research is the identification and determination of monitoring features in smoke-filled areas during the combustion of mixtures of organic compounds and polymers of various classes and solving the reverse problem—identification of organic compounds that were the source of ignition, using the acoustic method.

Promising is the search for the possibilities of the practical stasis of the established approach, both when navigating in a smoky space for special services officers, and when monitoring the situation in a statically installed environment.

Methods for detection and, in particular, acoustic flame extinguishing can have great potential for future applications, as exemplified in industrial plants, production halls, organic material warehouses, and even (due to specificity) in the space segment.

The number of additional environmental considerations when using the acoustic method in detectors, fire extinguishers, and monitors in smoke-filled areas will also be added to ensure the safety of personnel and the population in emergency situations.

## 5. Conclusions

In this paper, the ideas on the possibilities of using the acoustic method in fire extinguishing were further developed. The effect of combustion of various polymers on the acoustic parameters of the environment and the possibility of using them for monitoring the environment were shown, and the correlation between the extinguishing parameters and the distance to the ignition source was determined.

The results on the extinguishing of flames by the acoustic method are presented in the range of two frequencies of the extinguisher, i.e., 15 and 19 Hz, depending on the distance of the flame source from the extinguisher outlet. The research showed the effectiveness of acoustic waves emitted from the sound source in extinguishing flames of organic substances on the basis of paraffin wax. For the purpose of the experiment, a point flame source was used to unambiguously determine whether the flames were successfully extinguished. Generated, amplified, and focused sound waves of a low frequency and high or very high acoustic power allow the creation of local conditions in which the flame cannot sustain itself, as its continuous exposure in the acoustic field leads to its complete extinguishment without the need for chemicals. The power level required to extinguish the flames and the sound pressure level at the point where the flames were extinguished were analyzed. It was shown that with an increase in the distance between the waveguide outlet and the flame source, the required power necessary to extinguish the flames increases. Furthermore, there is a correlation between the increased power and the wave frequency, and the extinguishing effect depends on the frequency (as the frequency approaches that for which the minimum impedance was recorded, a lower value of power necessary to extinguish the flames is noted). The acoustic pressure at which the flames were successfully extinguished was in the range between 120 and 130 dB and, in practice, did not exceed a value of 128 dB. The pressure level decreased with the increasing distance between the waveguide outlet and the flame source. The diagrams confirmed these findings.

When studying the features of monitoring in smoke-filled areas during the combustion of organic compounds, correcting parameters were determined and correction factors were calculated for the development of acoustic action sensors: the angle of incidence, the wave resistance of the medium, the temperature, and the concentration of suspended particles. The dependence on the temperature gradient of the combustion products from various organic materials was determined, as well as the correcting correction value of the readings of the distance of the acoustic device, which is equal to +2.8 cm. Through the studies, it was determined that combustion products reduce the velocity of distribution of acoustic waves in the medium. The combustion of different materials, such as plastic, styrofoam, textile, gypsum plasterboard, wood, and motor oil, produces varying degrees of smoke and the concentration of combustion products. The loss of the velocity of acoustic waves in a medium filled with combustion products is shown to vary in the range from 8% to 25%, depending on the physical and chemical properties of the burning material.

The basis for the practical creation of an acoustic device, which differs from known ones by additional characteristics, namely, the ability to function effectively in conditions of high temperatures, dense smoke, and air pollution, has been developed.

Further ways of developing the application of the acoustic method in the extinguishing of organic compound fires and providing monitoring in the smoke-filled areas were noted.

The obtained results can become part of ecological–technical and ecological–economic solutions at the state and municipal levels, as well as a contribution to the national policy of individual states, aimed at supporting and developing ecological approaches while ensuring the goals of sustainable development.

## Figures and Tables

**Table 1:** Impact of the main products of combustion of organic substances on the environment and humans.

Combustion Product	Impact	References
Carbon monoxide (CO)	Causes shortness of breath, nausea, vomiting, dizziness, headache in humans, death is possible	[42]
Carbon dioxide (CO_2_)	Causes nausea, dizziness, blurred vision, loss of consciousness, respiratory acidosis	[43]
Ammonia ((NH_3_)	Irritates mucous membranes, causes coughing, lacrimation and cell necrosis, pulmonary edema, and death	[43]
Polycyclic aromatic hydrocarbons (PAH)(phenols, aldehydes, ketones, etc.)	Causes cancer in humans, genotoxicity	[44]
Hydrogen cyanide (HCN)	Paralyzes the respiratory system, causing tissue respiration disorders	[42]
Hydrogen chloride (HCl)	Causes acid rain. Causes irritation and inflammation of mucous membranes in humans	[42,44]
Phosphine (HF)	Causes irritation of mucous membranes and damage to the human nervous system, very toxic	[42]
Nitrogen oxide (NO)	Causes coughing, vomiting, shortness of breath, cyanosis, cardiovascular failure in humans	[42,44]
Sulfur dioxide (SO_2_)	Causes acid rain, negatively affects plants and animals. In humans, it causes spasm and swelling of the larynx	[44,45]
Nitrogen dioxide (NO_2_)	Causes acid rain. Irritates mucous membranes, causes respiratory diseases, coughing, wheezing, shortness of breath, pulmonary edema	[44,45]
Polychlorinated biphenyls (PCBs)	They are neurotoxicants, immunosuppressants, and carcinogens, affecting the thyroid gland and human reproductive functions	[46]
Volatile organic compounds (toluene, benzene, ethylbenzene and xylene, formaldehyde, etc.)	Cause irritation of human mucous membranes, contribute to the development of cancer. Toxic to the environment, contribute to global warming	[45,47]
Dioxins	They cause pigmentation and skin lesions, disorders of the immune, nervous, reproductive, and endocrine systems, and contribute to the development of cancer	[45]
Particulate matters	They irritate the mucous membranes and contribute to the development of respiratory diseases	[43,45]
Methane (CH_4_)	Does not cause direct harm to human health when inhaled, but is a major source of air pollution on a global scale	[48]

**Table 2:** Evaluation of the effect of combustion products from various materials on acoustic waves.

Material	Time to Reach Critical Concentration, s	Concentration of Combustion Products, mg/L	K	Loss of Velocity, %	Ambient Temperature, °C
Plastic	20	50	1.029	15	25
Styrofoam	11.2	50	1.035	20	
Textile	26.2	50	1.029	12	
Gypsum plasterboard	78.5	50	1.018	8	
Wood	32.7	50	1.03	25	
Paper	21.8	50	1.02	13	
Motor oil	15.7	50	1.04	25	

**Table 3:** Dependence of the temperature of the products of combustion of materials on the readings of the acoustic device.

№	Material	The Indicator of the Distance from the Device to the Obstacle at Different Temperatures, m					The Average Error Rate of Device Readings		Average Value of the Adjusting Parameter, m
		20 °C	40 °C	60 °C	80 °C	100 °C	Δ, m	Ϭ, %	
1	Motor oil	0.976	0.973	0.971	0.967	0.959	0.061	0.612	0.03
2.	Plastic	0.969	0.977	0.985	0.989	0.991	0.06	0.61	0.019
3	Styrofoam	0.971	0.965	0.961	0.968	0.982	0.07	0.745	0.035
4.	Textile	0.992	0.988	0.976	0.971	0.968	0.011	1.15	0.015
5.	Paper	0.963	0.968	0.969	0.971	0.973	0.03	0.27	0.034
6.	Gypsum plasterboard	0.979	0.981	0.983	0.985	0.988	0.1.72	1.74	0.017

## Data Availability

Data are contained within the article.
